# Supplementary material for: Postoperative acute respiratory dysfunction and the influence of antibiotics after acute type A aortic dissection surgery: A retrospective analysis
Source: PLoS One. 2021 Feb 11;16(2):e0246724. doi: 10.1371/journal.pone.0246724 (PMC7877985; doi:10.1371/journal.pone.0246724)
Supplement: Attachment — Submitted filename: Response to Reviewers_Revision_1.docx [file pone.0246724.s001.docx]

Dear editor and reviewers,

We would like to thank the academic editor Mrs. Balotta and the reviewers for their extensive and constructive review of our manuscript.
We have revised our paper and hope that our careful revision and our respective statements are satisfying. The following is a detailed response to the editors’ and reviewers’ comments.

**Editor:**

**Journal Requirements:**
When submitting your revision, we need you to address these additional requirements:

1. Please ensure that your manuscript meets PLOS ONE's style requirements, including those for file naming. The PLOS ONE style templates can be found at http://www.plosone.org/attachments/PLOSOne_formatting_sample_main_body.pdf and http://www.plosone.org/attachments/PLOSOne_formatting_sample_title_authors_affiliations.pdf

- Changes have been made in line 180, 300, 306, 313, 328, 342, 361, 381, 400, and 401.
- PACE has been used before the first submission.

2. We note that you have indicated that data from this study are available upon request. PLOS only allows data to be available upon request if there are legal or ethical restrictions on sharing data publicly. For information on unacceptable data access restrictions, please see http://journals.plos.org/plosone/s/data-availability#loc-unacceptable-data-access-restrictions.

In your revised cover letter, please address the following prompts:
a) If there are ethical or legal restrictions on sharing a de-identified data set, please explain them in detail (e.g., data contain potentially identifyingor sensitive patient information) and who has imposed them (e.g., an ethics committee). Please also provide contact information for a data access committee, ethics committee, or other institutional body to which data requests may be sent.
b) If there are no restrictions, please upload the minimal anonymized data set necessary to replicate your study findings as either Supporting Information files or to a stable, public repository and provide us with the relevant URLs, DOIs, or accession numbers. Please see http://www.bmj.com/content/340/bmj.c181.long for guidelines on how to de-identify and prepare clinical data for publication. For a list of acceptable repositories, please see http://journals.plos.org/plosone/s/data-availability#loc-recommended-repositories.

- As we pointed out, the data are legally restricted and cannot be shared publicly. They are available any time upon request by contacting our Department at the University of Regensburg. The head of our research laboratory, Dr. Michael Gruber takes care of a safe data backup.
- https://www.ukr.de/kliniken-institute/Anaesthesiologie/Klinikteam___Mitarbeiter/Forschungslabor/index.php

**Reviewer #1:** Tahnk you for your paper.
The paper is interesting because it consider the impact of our antibiotic on the outcome of our patients, despite that in my opinion I think the use of broad spectrumantibiotic is influence non only by the lung status but also from the clinical analysis.
Patients who are going clinically worst need to be covered by broad spectrum non only because the lung problem.
I don't understand how patients who develop ARF can die less then who don't, it might be the selection of ARF definition.

- Yes you are right, we were also surprised by our results. There might be a selection bias due to the ARD definition. However, other factors might indepently influence the outcome. For example, a recent meta-analysis showed that the body mass index can predict the clinical outcomes for patients with acute respiratory distress syndrome. Ni et al. showed that obesity and morbid obesity was associated with a lower mortality in patients with ARDS. We found a higher BMI in our ARD group. Accordingly, this might have influenced our outcome with a lower mortality. With regard to your very important annotation, we added the following sentence in line 218: “But it should not be neglected that it could also be a selection bias due to the ARD definition.” Furthermore, we revised our sentence in line 274: “Second, the different ARD definitions used in other studies make it difficult to compare their results with each other and with our results; and it might have had an impact on our results.”

**Reviewer #2:** The Authors investigated the incidence, risk factors and impact on clinical outcomes of acute respiratory failure after surgery for acute type A aortic dissection. The retrospective cohort spans from 2008 to 2017.
I only have a few comments:
- did surgical techniques and cerebral protection techniques change during the study period?

- This is a very important annotation. It might be comprehensible that over the time period of 9 years a change in the surgical technique and/or cerebral protection might have occurred. Unfortunately, this was not well documented.
- As we totally agree, we therefore changed the manuscript (line 282): “Fourth, we cannot clarify if the operation technique and cerebral protection has changed over the years. This might have had an impact on the outcome of the patients.”

- most patients were managed with moderate hypothermia. Did this change over time? If so, did this impact on transfusional requirements? Do the Authors believe that this had an impact on respiratory complications?

- The management of hypothermia did not change over the time. Therefore, transfusional requirements were not different. These results are listed in table 2. To respond to this important annotation, we changed our manuscript (line 66): “The management of hypothermia did not change over the time.”

- did the incidence of postoperative respiratoryfailure change along the study period?

- This is a very interesting question! We have looked at our data. We found a different incidence of postoperative respiratory failure per year. However, we did not see a trend over the time. Therefore, we changed our manuscript (line 127): “The incidence of postoperative respiratory failure changed from year to year; however, a trend along the study period could not be detected.”
