# Supplementary material for: Postoperative acute respiratory dysfunction and the influence of antibiotics after acute type A aortic dissection surgery: A retrospective analysis
Source: PLoS One. 2021 Feb 11;16(2):e0246724. doi: 10.1371/journal.pone.0246724 (PMC7877985; doi:10.1371/journal.pone.0246724)
Supplement: Attachment — Submitted filename: Response to Reviewers_Revision_2.docx [file pone.0246724.s003.docx]

Dear editor,

We would like to thank the academic editor Mr. Zivkovic for his extensive and constructive review of our manuscript. We have not found any comments from reviewers and are therefore only guided by the comments from the Academic editor.
We have revised our paper and hope that our careful revision and our respective statements are satisfying. The following is a detailed response to the editors’ comments. Our line specifications refer to the “revised manuscript with track changes”.

**Editor:**

**Journal Requirements:**

1. Please provide additional details regarding participant consent. In the Methods section, please ensure that you have specified (1) whether consent was informed and (2) what type you obtained (for instance, written or verbal). If your study included minors, state whether youobtained consent from parents or guardians. If the need for consent was waived by the ethics committee, please include this information.

- With regard to your very important annotation, we added the following sentence in line 62: “Due to the design of the study an informed consent was not necessary.”

Once you have amended this/these statement(s) in the Methods section of the manuscript, please add the same text to the “Ethics Statement” field of the submission form (via “Edit Submission”).
For additional information about PLOS ONE ethical requirements for human subjects research, please refer to http://journals.plos.org/plosone/s/submission-guidelines#loc-human-subjects-research

- The Ethics Statement field of the submission form has been changed.

**Additional Editor Comments (if provided):**

The manuscript has merits to be accepted for the publication, however, I would like to point out the following matters:

Please consider addressing the following major points:

1. It is not clear which criteria were used to make the decision on using extended/broad spectrum antibiotics. Please describe the criteria. In case the criteria are not available/ the decision was made based on the attending clinician’s discretion, this, as well, needs to be described.

- Yes, the decision was made by the physician in charge. Line 90: “Patients received either narrow-spectrum or broad-spectrum antibiotics depending on the physician in charge.”
  To make the sentence more prominent, it was moved to the end of the section “Anesthetic and ICU management”.

2. Please consider including only two patient groups: patients with acute respiratory dysfunction (ARD) and those without (NON-ARD). Furthermore, I would suggest describing the profile and the percentage of patients receiving narrow- or broad spectrum antibiotics within these two groups. Additional subdivision of the patients into the further two groups (based on narrow/broad spectrum antibiotics) could place the results out of focus. The main finding, that the type of the antibiotic regime did not correlate with the occurrence of the ARD, could be better emphasized if the authors focus on the two patient groups and describe the findings accordingly.

- Thank you for the valuable comment. We completely agree with you and have therefore made some changes to focus more on the groups NON-ARD and ARD.
  Line 24: “All patients received either narrow- or broad-spectrum antibiotics.”
  Line 32: “The incidence of pneumonia was independent of the antibiotic treatment regime (*p* = .391). Renal and neurological complication rate was higher in patients treated with broad-spectrum antibiotic.”
  We have deleted the paragraph Classification of antibiotic groups and inserted therefore the sentence (line 90): “Patients received either narrow-spectrum or broad-spectrum antibiotics depending on the physician in charge. Narrow-spectrum antibiotic was normally a one day course of cefuroxime and broad-spectrum antibiotic was normally piperacillin/tazobactam for several days. Both were started within the first 24 hours after admission.”
  Lines 167-179 have been rewritten: “In the NON-ARD group 43% of the patients (n = 39) were treated with a narrow-spectrum antibiotic and 57% (n = 51) […]”
  Lines 259-263 and 267-271 have also been rewritten.
  The sentence in line 324/325 has been deleted.
  Supporting information S1 and S2 table has been removed.
  Figure 3 has been changed into a new figur.

Minor points:
1. Line 23: please, introduce (define) the PF ratio: “PaO2/FiO2≤ 200 mmHg (PF ratio) within…”

- The sentence has been corrected: “ARD was defined as PaO_2_/FiO_2_ ≤ 200 mmHg (PF ratio) within 48 hours after surgery.”

2. Line 29: please, stayconsistent in describing patient groups: first ARD, followed by the NON-ARD (or, alternatively, the other way around), but, please, keep consistency throughout the abstract text.

- The abstract has been revised and changes have been made.

3. Line 51: please, consider rephrasing the term “certain”.

- This has been changed (line 54).

4. Line 61: Please, rephrase the following: “especially”

- “Especially” has been changed into “particularly” (line 65).

5. Line 64: please, remove “commonly”.

- We removed the word “commonly” (line 68).

6. Line 66: please, consider rephrasing the following sentence: “For cerebral protection hypothermia was induced”

- Thank you, the sentence has been rewritten: “Hypothermia was induced for cerebral protection.” (line 70).

7. Line 77: please, remove “typical”

- The word “typical” has been removed (line 84).

8. Line 78: please, consider rephrasing the following: “The main criteria for extubation were… the level of consciousness… the ability to follow instructions…” The criterion for the extubation is, presumably, the sufficient level of consciousness (RASS? GCS?), which includes the ability to follow instructions.

- You are absolutely right. In our intensive care unit GCS and RASS is used to determine the level of consciousness. The sentence has been corrected in line 84: “The main criteria for extubation were adequate gas exchange, the level of consciousness (for example GCS and RASS), normothermia, hemodynamic stability, a positive cough reflex and no significant bleeding.”

9. Line 104: please use “skewed distribution”instead of “skewed distributed”

- This error has been corrected in line 118.

10. Line 127: it is not clear what was significantly different. Please, rephrase this sentence and include the statistics. Consider pointing out whether the observed difference regards to the groups or the time points of the measurements.

- This is a very important annotation. Therefore, we changed our manuscript (line 140): “Each PF ratio between the ARD and NON-ARD groups was significantly different from the preoperative admission until the seventh postoperative day.”

11. Line 149: Please rephrase the term “slightly”

- The word has been changed into “a few” (line 162).

12. Line 172: please, check the sentence: “… than those in the patients treated with…”

- Sentences have been corrected (line 27, line 206, line 216, line 239 and line 245).

13. Line 229: It is not clear whether authors refer to the findings of their study or the current literature regarding the term “at the moment”. Please rephrase.

- The phrase “in the literature” has been added (line 259).

14. Line 238: please consider rephrasing “… than those of patients who…”

- The sentence in line 271 has been removed. The sentence in line 302 has been corrected.

15. Line: 248: “because this was not the focus of our study”. Please rephrase. The reason for not knowing which patients experienced malperfusion might be the fact that the records regarding this information aremissing, however, not the fact that the it is “not the focus of the study”.

- This is absolutely correct. The sentence has been corrected into (line 282): “(…) because this was not documented.”

16. Line 264: please change “did not led” into “did not lead”

- This error has been corrected (line 298).

17. Line 266: please change “assumingly” into “presumably”. Moreover, consider discussing the option that patients who received broad spectrum antibiotics showed clinically more severe illness than those receiving narrow spectrum antibiotics. Do authors have access to disease severity scores obtained at the ICU (SOFA, APACHE, SAPS, TISS)?

- “Assumingly” has been changed into “presumably” (line 300).
  Unfortunately this was not sufficiently documented.

18. Lines 266 and 268: please, consider rephrasing the following: can antibiotic therapy be “more effective”/ “less intensified”?

- Thank you. We therefore changed the manuscript (line 299): “Similarly, patients in the present study who received a broad-spectrum, and presumably more effective, antibiotic therapy did not have better postoperative outcomes and had more nephrological and neurological complications than patients who received a narrow-spectrum antibiotic treatment.”

19. Line 269: please consider rephrasing the first sentence.

- The sentence has been rewritten (line 304): ”The present study has some potential limitations due to its retrospective design.”

20. Line 274: consider using the term: multi-centre study.

- The word “multi-centre study” has been used (line 309).

21. Line 286: please check: “than that in patients”

- This has been corrected (line 323).

22. Line 287: please, remove/change the term “appear”

- The sentence has been deleted (line 325).

23. Line 289: please check: “than those in patients”

- Thank you. This has been corrected (line 326).

**Reviewers' comments:**

**/**
